# Atypical presentations of coronavirus disease 2019 (COVID-19) from onset to readmission

**DOI:** 10.1186/s12879-020-05751-8

**Published:** 2021-01-29

**Authors:** Zhiqi Yang, Xiaofeng Chen, Ruibin Huang, Shengkai Li, Daiying Lin, Zhijian Yang, Hongfu Sun, Guorui Liu, Jinming Qiu, Yanyan Tang, Jianning Xiao, Yuting Liao, Xianheng Wu, Renhua Wu, Xiangguang Chen, Zhuozhi Dai

**Affiliations:** 1 Department of Radiology, Meizhou People’s Hospital, Meizhou, Guangdong 514031 People’s Republic of China; 2 Department of Radiology, First Affiliated Hospital, Shantou University Medical College, Shantou, Guangdong 515041 People’s Republic of China; 3 Department of Radiology, Huizhou Municipal Central Hospital, Huizhou, 516001 Guangdong China; 4 Department of Radiology, Shantou Central Hospital, Shantou, Guangdong 515041 People’s Republic of China; 5 Department of Radiology, Yongzhou People’s Hospital, Yongzhou, Hunan 425006 People’s Republic of China; 6 School of Information Technology and Electrical Engineering, University of Queensland, Brisbane, Queensland 4072 Australia; 7 Department of Radiology, 2nd Affiliated Hospital, Shantou University Medical College, Shantou, Guangdong 515000 People’s Republic of China; 8 GE Healthcare, Guangzhou, 510623 China

**Keywords:** COVID-19, Asymptomatic, Re-detectable positive, CT imaging negative, Features

## Abstract

**Background:**

To investigate the CT imaging and clinical features of three atypical presentations of coronavirus disease 2019 (COVID-19), namely (1) asymptomatic, (2) CT imaging-negative, and (3) re-detectable positive (RP), during all disease stages.

**Methods:**

A consecutive cohort of 79 COVID-19 patients was retrospectively recruited from five independent institutions. For each presentation type, all patients were classified into atypical vs. typical groups (i.e., asymptomatic vs.symptomatic, CT imaging-negative vs. CT imaging-positive, and RP and non-RP,respectively). The chi-square test, Student’s *t* test, and Kruskal-Wallis *H* test were performed to compare CT imaging and clinical features of atypical vs. typical patients for all three presentation categories.

**Results:**

In our COVID-19 cohort, we found 12.7% asymptomatic patients, 13.9% CT imaging-negative patients, and 8.9% RP patients. The asymptomatic patients had fewer hospitalization days (*P*=0.043), lower total scores for bilateral lung involvement (*P*< 0.001), and fewer ground-glass opacities (GGOs) in the peripheral area (*P*< 0.001) than symptomatic patients. The CT imaging-negative patients were younger (*P*=0.002), had a higher lymphocyte count (*P*=0.038), had a higher lymphocyte rate (*P*=0.008), and had more asymptomatic infections (*P*=0.002) than the CT imaging-positive patients. The RP patients with moderate COVID-19 had lower total scores of for bilateral lung involvement (*P*=0.030) and a smaller portion of the left lung affected (*P*=0.024) than non-RP patients. Compared to their first hospitalization, RP patients had a shorter hospitalization period (*P*< 0.001) and fewer days from the onset of illness to last RNA negative conversion (*P*< 0.001) at readmission.

**Conclusions:**

Significant CT imaging and clinical feature differences were found between atypical and typical COVID-19 patients for all three atypical presentation categories investigated in this study, which may help provide complementary information for the effective management of COVID-19.

**Supplementary Information:**

The online version contains supplementary material available at 10.1186/s12879-020-05751-8.

## Background

The coronavirus disease 2019 (COVID-19) pandemic is a global crisis and has caused hundreds of thousands of people to die as of June 8, 2020 [1]. Typical COVID-19 patients present with fever, cough, fatigue, normal white blood cell count (WBC), lower lymphocyte count, and pure or mixed ground-glass opacity (GGO) in the subpleural region [2–4]. However, prevention and control practices found that some patients did not present with these typical manifestations [5–7]. These patients challenge the prevention and control system and increase the risk of the COVID-19 spread. Identifying the atypical presentations is helpful for a comprehensive understanding of COVID-19 and infection control.

We categorized all patients into three atypical presentations of COVID-19: (1) asymptomatic, (2) CT imaging-negative, and (3) re-detectable positive (RP). In the early stages of COVID-19, asymptomatic and CT imaging-negative patients were easily overlooked and became hidden sources of infection. As an increasing number of patients are discharged, RP patients have gradually become the focus of attention. Recent studies have shown evidence of human-to-human transmission from asymptomatic patients [8–11]. CT imaging-negative and RP patients have also been reported in multiple studies [7, 12, 13]. However, limited data are available to provide a clear picture of the imaging and clinical features in all three types of atypical patients.

In this study, we aimed to identify the differences in CT imaging and clinical features from onset to discharge between typical and atypical patients with COVID-19 based on multicentre data. These data may help provide complementary information for more effective management of COVID-19.

## Materials and methods

### Patients

Ethical approval by the institutional review boards was obtained for this retrospective study, and the need to obtain informed consent was waived. From January 1 to April 22,020, a total of 79 consecutive patients with SARS-CoV-2 infection confirmed by real-time reverse transcription polymerase chain reaction (RT-PCR) were enrolled from 5 independent hospitals. Seventy-three patients were reported in our previous study [2, 3]. Of all patients, including 27 patients from Huizhou City, 27 from Shantou City, 15 from Yongzhou City and 10 from Meizhou City, the mean age was 41.8 years (range: 3~69 years). Overall, 69 patients were symptomatic (mean age: 42.2 years; range: 3~67 years) and 10 were asymptomatic (mean age: 38.8 years; range: 16~69 years); additionally, 11 patients were CT imaging negative (mean age: 29.8 years; range: 3~48 years), and 68 were CT imaging positive (mean age: 43.7 years; range: 14~69 years).

**Fig. 1:**
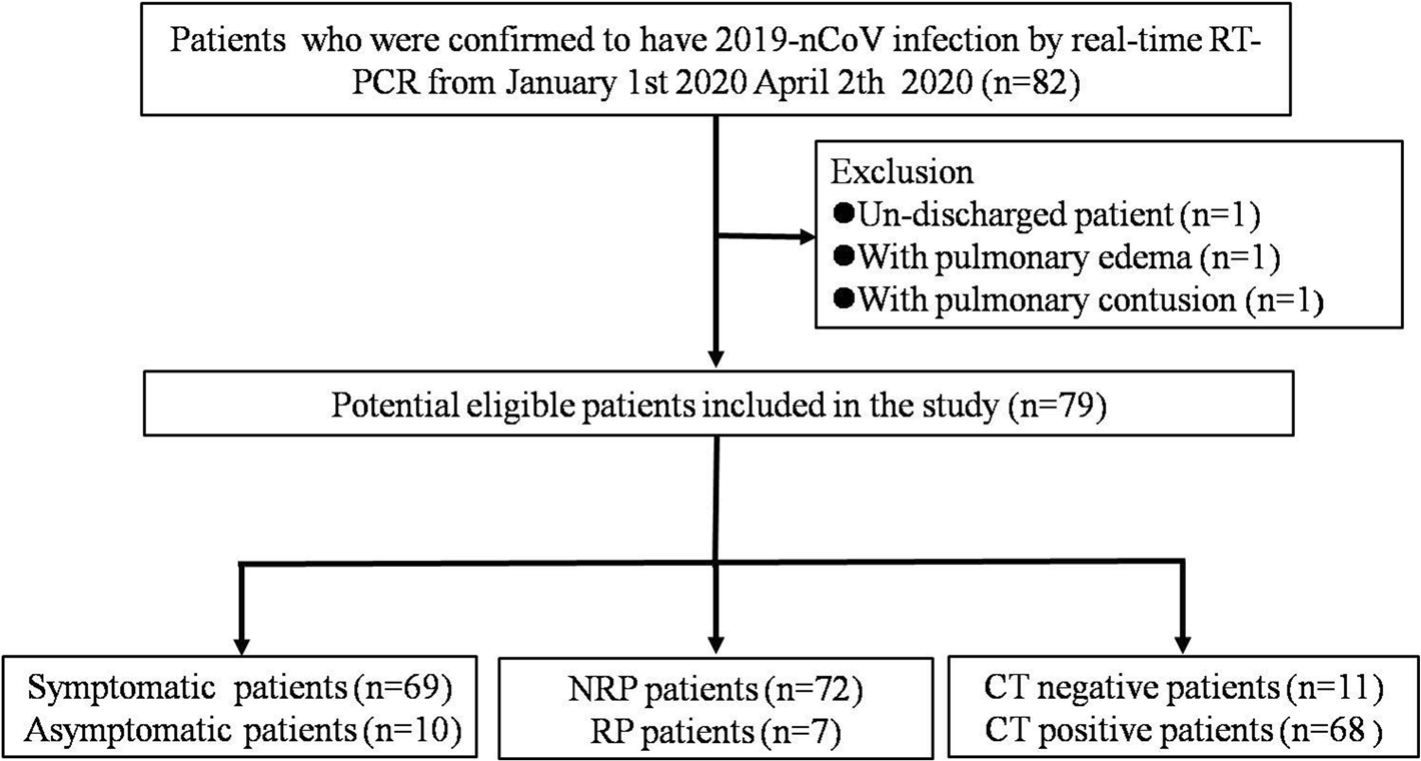
Flow chart of patient selection and exclusion

According to the sixth edition guideline published by the National Health Commission of China [14], all discharged COVID-19 patients should be isolated and observed for 2 weeks. SARS-CoV-2 RNA detection from digestive and respiratory sites was performed weekly. The RP patients were readmitted to the hospital for further medical observation, and close contacts were also followed-up. As of April 30, 2020, all of the patients were followed up for at least 14 days, and 7 patients (8.9%) were RP, including 4 patients from Huizhou City, 2 from Shantou City, and 1 from Meizhou City, with a mean age of 41.0 years old (range: 14~66 years). Among the RP patients, 4 were men (mean age: 37.8 years; range: 27~61 years), and 3 were women (mean age: 45.3 years; range: 14~66 years). Figure 1 shows the patient recruitment pathway of the symptomatic and asymptomatic infection patients, non-RP (NRP) and RP patients, CT imaging-negative and CT imaging-positive patients, along with the inclusion and exclusion criteria.

### Clinical data collection

The initial clinical data from the first hospitalization and readmission, including age, sex, course of the disease, clinical symptoms, clinical type of COVID-19, hospitalization days, days from the onset of illness to the last RNA negative conversion, WBC count, lymphocyte count, lymphocyte ratio, neutrophil count and neutrophil ratio were collected. The threshold values for WBC count, lymphocyte count, lymphocyte ratio, neutrophil count and the neutrophil ratio were 3.5~9.5× 10^9^/L, 1.1~3.2× 10^9^/L, 20.0~50.0%,1.8~6.3 × 10^9^/L, and 40.0~75.0%, respectively, according to the normal range used at the individual hospital.

### CT image acquisition and review

Non-contrast-enhanced chest CT imaging data were obtained from multiple hospitals using different CT systems, including GE CT Discovery 750 HD (General Electric, US), SCENARIA 64 CT (Hitachi Medical, Japan), PHILIPS Ingenuity CT (PHILIPS, Netherlands), and Siemens SOMATOM Definition AS (Siemens, Germany) systems. All images were reconstructed into 1 mm slices with a slice interval of 0.8 mm. The detailed acquisition parameters are summarized in the supplementary material (Table E1).

The initial CT imaging from the first hospitalization for all patients and initial CT imaging at readmission for RP patients were evaluated. A total of 25 quantitative and 18 qualitative imaging features were extracted for analysis. The descriptions of the CT imaging features are listed in the supplementary material (Table E2 and Table E3). For the extraction of CT qualitative and quantitative imaging features, two senior radiologists (Z.Y. and X.C., more than 10 years of experience) reached a consensus and were blinded to the clinical and laboratory findings. A lesion in the outer third of the lung was defined as peripheral, and a lesion in the inner two-thirds was defined as central [2]. The classification of the lesion size was based on a previous study [2]. The progression of the lesion within each lung lobe was evaluated by scoring each lobe from 0 to 4 [2], corresponding to normal, 1% ~ 25% infection, 26%~ 50% infection, 51%~ 75% infection and more than 75% infection, respectively. The scores were combined for all 5 lobes to provide a total score.

### Statistical analysis

For quantitative imaging features and qualitative imaging features, we use interclass correlation coefficients (ICC) and Cohen’s Kappa to analyze the consistency of the two radiologists, respectively. The CT imaging and clinical features were compared between symptomatic and asymptomatic infection groups, NRP and RP patient groups, and CT imaging-negative and CT imaging-positive groups by using the chi-square test Fisher’ *t* test, the Student’s *t* test, and Kruskal-Wallis *H* test. All statistical analyses were performed with R (version 3.6.4). All statistical tests were two-sided, and differences were considered significant at *P*< 0.05.

## Results

### Baseline characteristics of COVID-19 patients

**Fig. 2:**
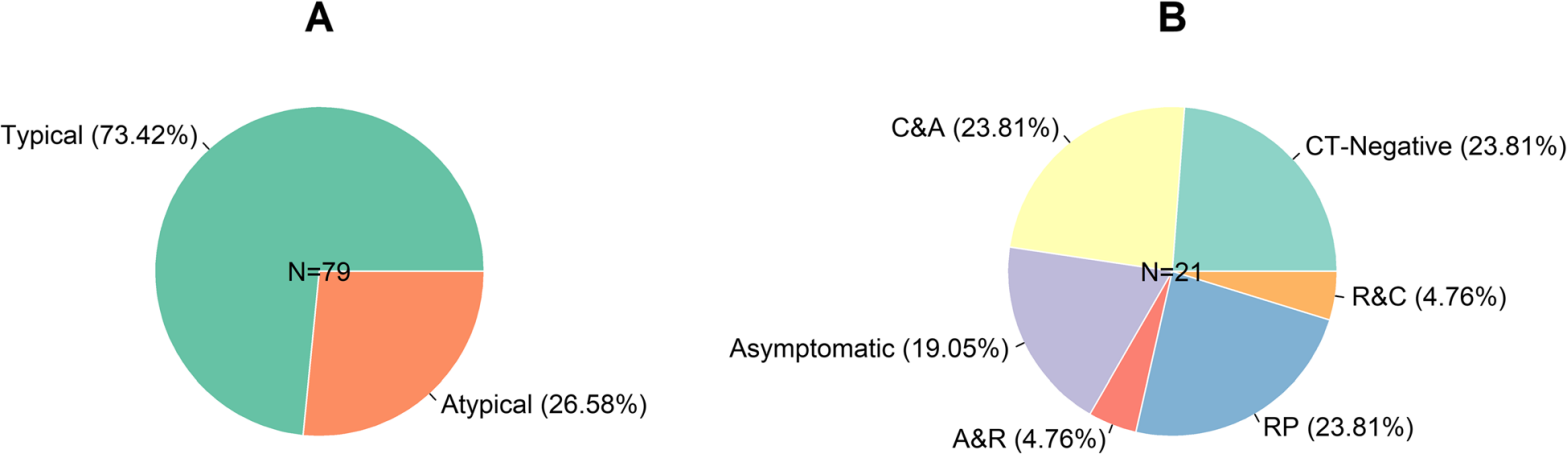
Pie chart of COVID-19 patients. **a**: typical and atypical percentage of COVID-19 patients. **b**: CT-negative patients (C), asymptomatic patients (A), RP patients (R), and the combined percentage in atypical COVID-19 patients

In our COVID-19 patients, 58 (73.42%) had typical COVID-19, and 21 (26.58%) had atypical COVID-19. In the atypical COVID-19 group, 5 (23.81%) were CT imaging negative, 4 (19.05%) were asymptomatic, 5 (23.81%) were RP, 5 (23.81%) were both CT imaging negative and asymptomatic, and 1 (4.76%) was both asymptomatic and RP, 1 (4.76%) was both RP and CT imaging negative (Fig. 2).

### Clinical and CT imaging feature comparison between the symptomatic and asymptomatic infection groups

Consistency analysis result show than the ICC value of quantitative imaging features and kappa value of qualitative imaging features were greater than 0.95.The clinical features of the symptomatic and asymptomatic infection patients are shown in Table 1. A total of 50.0% of asymptomatic infection patients had mild COVID-19 pneumonia. None of the asymptomatic infection patients developed severe COVID-19 pneumonia or died during follow-up. Compared to symptomatic infection patients, asymptomatic infection patients had a shorter hospitalization stay (*P*=0.043). There was no significant difference in sex, age, rate of re-detectable RNA after discharge, days of last RNA negative conversion since the onset of illness, WBC count, lymphocyte count, lymphocyte rate, neutrophil countor and neutrophil rate between symptomatic and asymptomatic infection patients.

**Table 1:** Clinical features of symptomatic and asymptomatic COVID-19 patients

Features	Symptomatic (*n*=69)	Asymptomatic (*n*=10)	*P*-value
Sex			0.962^a^
Male^#^	38(55.1%)	6(60.0%)	
Female^#^	31(44.9%)	4(40.0%)	
Age	42.2±13.9	38.8±17.1	0.484^b^
Course of disease	2.51±2.63	2.40±2.37	0.903^b^
Clinical type			0.001^c^*
Mild^#^	6(8.7%)	5(50.0%)	
Moderate^#^	56(81.2%)	5(50.0%)	
Severe^#^	7(10.1%)	0(0.0%)	
Re-detectable of RNA after discharged			1.00^a^
Negative^#^	63(91.3%)	9(90.0%)	
Positive^#^	6(8.70%)	1(10.0%)	
Hospitalization days	16.6±6.71	12.0±5.85	0.043^b^*
Days of last RNA negative-conversion	13.0±6.56	10.4±5.93	0.237^b^
Days of last RNA negative-conversion category			0.319^c^
< 7 days^#^	9(13.0%)	1(10.0%)	
7 days~ 14 days^#^	35(50.7%)	8(80.0%)	
14 days~ 21 days^#^	22(31.9%)	0(0.0%)	
> 21 days^#^	3(4.4%)	1(10.0%)	
WBC count (×10^9^/L)	5.51±2.50	4.95±1.90	0.502^b^
Lymphocyte count	1.30±0.87	1.37±0.39	0.813^b^
Lymphocyte rate (%)	24.4±11.2	30.7±11.7	0.100^b^
Neutrophil count(×10^9^/L)	3.70±2.27	3.15±1.89	0.469^b^
Neutrophil rate (%)	65.0±14.8	60.2±11.6	0.328^b^

Note: *Data with statistical significance. ^#^Results are measurements with corresponding ratio in parentheses, and the remainder results are mean value with standard deviation. *P*^a^: chi square test, *P*^b^: student’s *t* test, *P*^c^: Kruskal-Wallis *H* test, *WBC* White blood cell

**Fig. 3:**
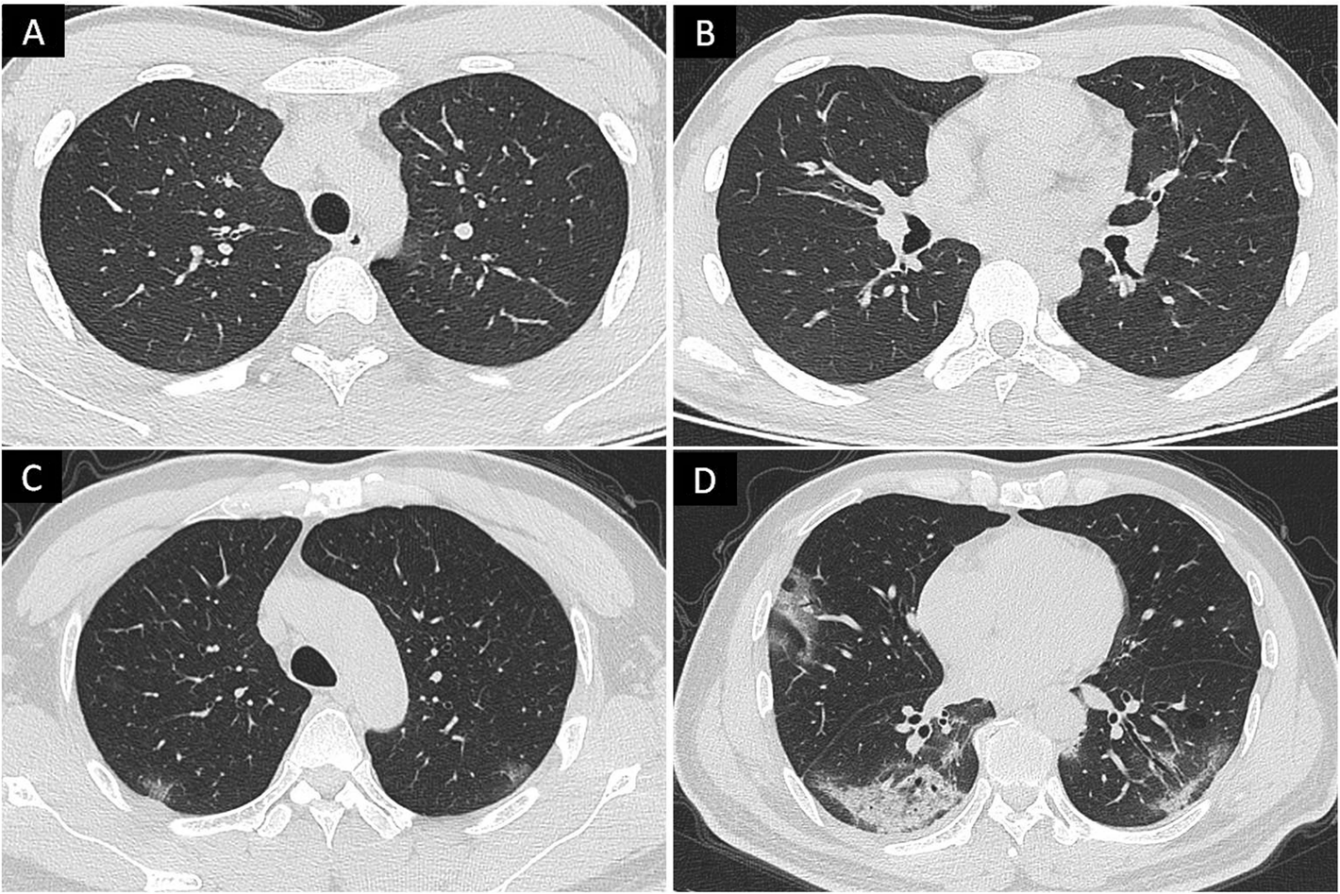
3**a-**3**b**: Asymptomatic infection with moderate COVID-19 patient. There are one pure ground-glass opacities under pleural of right lung upper lobe. The remaining double lung did not see obvious abnormalities. The total scores of involved zones in the bilateral lung is 1. 3**c**-3**d**: Symptomatic infection with moderate COVID-19 patient. There are multiple pure and mixed ground-glass opacities under double lung pleural. The total scores of involved zones in the bilateral lung is 6

The CT imaging features of the symptomatic and asymptomatic infection patients are shown in Table E4, and those imaging features with significant differences are presented in Table 2. Representative CT images from symptomatic and asymptomatic infection patients are shown in Fig. 3. A total of 50.0% asymptomatic infection patients had normal chest CT imaging. Compared to symptomatic infection patients, asymptomatic infection patients had a smaller total number of pure GGOs in the peripheral area (*P*< 0.001), mixed GGOs in the peripheral area (*P*< 0.001), total number of lesions in the peripheral area (*P*< 0.001), proportion of the left lobe affected (*P*=0.005), and proportion of the right lobe affected (*P*=0.001), as well as lower total bilateral lung scores (*P*< 0.001), and lower total right lung scores (*P*< 0.001) and left lung scores (*P*< 0.001). In addition, the negative rates of pure GGOs (60.0%), pure GGOs in peripheral areas (70.0%), mixed GGOs (70.0%), and mixed GGOs in peripheral areas (70.0%) were more pronounced in asymptomatic infection patients (all *P*< 0.05).

**Table 2:** CT features of symptomatic and asymptomatic COVID-19 patients

Features	Symptomatic (*n*=69)	Asymptomatic (*n*=10)	*P*-value
Overall condition of lesions			< 0.001^c^*
Normal^#^	6(8.7%)	5(50.0%)	
Single^#^	4(5.8%)	2(20.0%)	
Multiple^#^	59(85.5%)	3(30.0%)	
Total number of GGO inperipheral area			
Pure GGO	5.36±7.28	0.90±2.18	< 0.001^b^*
Mixed GGO	4.83±7.05	0.50±0.85	< 0.001^b^*
Total number of lesions			
Peripheral area	11.6±13.8	1.40±2.22	< 0.001^b^*
Central area	1.81±4.41	0.80±1.93	0.478^b^
Total scores of involved lung zones			
Bilateral lung	5.26±4.01	1.30±1.83	< 0.001^b^*
Right lung	3.01±2.45	0.80±1.23	< 0.001^b^*
Left lung	2.25±1.86	0.50±0.71	< 0.001^b^*
Number of lobes affected			
Right lung	1.93±1.15	0.80±1.23	0.005^b^*
Left lung	1.39±0.79	0.50±0.71	0.001^b^*
Total scores of bilateral lung category			0.002^a^*
< 3 scores^#^	18(26.1%)	8(80.0%)	
≥3 scores^#^	51(73.9%)	2(20.0%)	
Pure GGO category			0.021^a^*
Negative^#^	14(20.3%)	6(60.0%)	
Positive^#^	55(79.7%)	4(40.0%)	
Pure GGO in peripheral area category			0.003^a*^
Negative^#^	14(20.3%)	7(70.0%)	
Positive^#^	55(79.7%)	3(30.0%)	
Mixed GGO category			0.003^a^*
Negative^#^	14(20.3%)	7(70.0%)	
Positive^#^	55(79.7%)	3(30.0%)	
Mixed GGO in peripheral area category			0.008^a^*
Negative^#^	16(23.2%)	7(70.0%)	
Positive^#^	53(76.8%)	3(30.0%)	

Note: *Data with statistical significance. ^#^Results are measurements with the corresponding ratio in parentheses, and the remainingresults are mean value with standard deviation. *P*^a^: chi-square test, *P*^b^: student’s *t*-test, *P*^c^: Kruskal-Wallis *H* test, *GGO* Ground-glass opacity

### Clinical feature comparison between the CT-negative and CT-positive patient groups

The clinical features of the CT imaging-negative and CT imaging-positive patients are listed in Table 3. Compared to CT imaging-positive patients, CT imaging -negative patients were younger and had higher lymphocyte counts, higher lymphocyte rates, and more asymptomatic infections (45.5%). In addition, CT imaging-negative patients presented with more asymptomatic infections than the CT imaging-positive patients (*P*< 0.001). There was no significant difference in sex, course of the disease, hospitalization days, or days since last RNA negative conversion between the CT-negative and CT-positive patients.

**Table 3:** Clinical features of CT negativeand positive patients with COVID-19

Features	CT- negative (*n*=11)	CT-positive (*n*=68)	*P*-value
Sex			0.807^a^
Male^#^	6(54.5%)	38(55.9%)	
Female^#^	5(45.5%)	30(44.1%)	
Age	29.8±11.9	43.7±13.8	0.002^b^*
Course of disease	2.09±2.26	2.56±2.64	0.580^b^
Clinical types			< 0.001^c^*
Mild^#^	11(100%)	0(0.0%)	
Moderate^#^	0(0.0%)	61(89.7%)	
Severe^#^	0(0.0%)	7(10.3%)	
Symptoms			0.002^a^*
Symptomatic^#^	6(54.5%)	63(92.6%)	
Asymptomatic^#^	5(45.5%)	5(7.4%)	
Re-detectable of RNA after discharged			1.00^a^
Negative^#^	10(90.9%)	62(91.2%)	
Positive^#^	1(9.1%)	6(8.8%)	
Hospitalization days	12.6±6.01	16.6±6.74	0.065^b^
Days of last RNA negative-conversion	9.73±5.80	13.2±6.53	0.105^b^
Days of last RNA negative-conversion category			0.056^c^
< 7 days^#^	3(27.3%)	7(10.3%)	
7 days to 14 days^#^	7(63.6%)	36(52.9%)	
14 days to 21 days^#^	0(0.0%)	22(32.4%)	
> 21 days^#^	1(9.1%)	3(4.4%)	
WBC count (×10^9^/L)	6.22±2.24	5.31±2.45	0.249^b^
Lymphocyte count	2.23±1.48	1.16±0.55	0.038^b^*
Lymphocyte rate (%)	33.5±13.2	23.8±10.5	0.008^b^*
Neutrophil count(×10^9^/L)	3.59±1.82	3.64±2.30	0.955^b^
Neutrophil rate (%)	57.1±13.9	65.6±14.3	0.070^b^

Note:*Data with statistical significance. ^#^Results are measurements with corresponding ratio in parentheses, and the remainder results are mean value with standard deviation. *P*^a^: chi square test, *P*^b^: student’s *t* test, *P*^c^: Kruskal-Wallis *H* test, *WBC* White blood cell

### Clinical and CT imaging feature comparison between the NRP and RP patient groups

As of April 232,020, 79 patients were followed up for at least 14 days, and 7 patients were re-detected as positive for SARS-CoV-2 during follow-up. The clinical features of the NRP and RP patients are shown in Table 4. Among the RP patients, 6 were moderate COVID-19 patients, and 1 was a mild COVID-19 patient. None of patients developed severe COVID-19 pneumonia during hospitalization. Most RP patients had clinical symptoms (85.7%). There was no significant difference in sex, age, course of the disease, hospitalization days, days since last RNA negative conversion,or clinical laboratory findings between the NRP and RP patients.

**Table 4:** Clinical features of RP and NRP patients with COVID-19

Features	NRP patients (*n*=72)	RP patients(n=7)	*P*-value
Sex			
Male^#^	40(55. 6%)	4(57.1%)	0.751^a^
Female^#^	32(44.4%)	3(42.9%)	
Age	41.9±13.8	41.0±19.8	0.880^b^
Course of disease	2.50±2.58	2.43±2.82	0.950^b^
Clinical types			0.604^c^
Mild^#^	10(13.9%)	1(14.3%)	
Moderate^#^	55(76.4%)	6(85.7%)	
Severe^#^	7(9.70%)	0(0.0%)	
Symptoms			1.00^a^
Symptomatic^#^	63(87.5%)	6(85.7%)	
Asymptomatic^#^	9(12.5%)	1(14.3%)	
Hospitalization days	15.8±6.86	18.3±5.35	0.357^b^
Days of last RNA negative-conversion	12.4±6.58	15.3±5.38	0.270^b^
Days of last RNA negative-conversion category			0.096^c^
< 7 days^#^	10(13.9%)	0(0.0%)	
7 days to 14 days^#^	40(55.6%)	3(42.8%)	
14 days to 21 days^#^	19(26.4%)	3(42.9%)	
> 21 days^#^	3(4.1%)	1(14.3%)	
WBC count (× 10^9^/L)	5.28±2.30	7.07±3.29	0.062^b^
Lymphocyte count	1.32±0.86	1.22±0.38	0.763^b^
Lymphocyte rate (%)	25.6±11.4	21.0±10.4	0.313^b^
Neutrophil count(×10^9^/L)	3.48±2.01	5.14±3.23	0.228^b^
Neutrophil rate (%)	64.4±14.3	63.9±17.6	0.931^b^

Note:*Data with statistical significance. ^#^Results are measurements with corresponding ratio in parentheses, and the remainder results are mean value with standard deviation. *P*^a^: chi square test, *P*^b^: student’s *t* test, *P*^c^: Kruskal-Wallis *H* test, *WBC* White blood cell

**Fig. 4:**
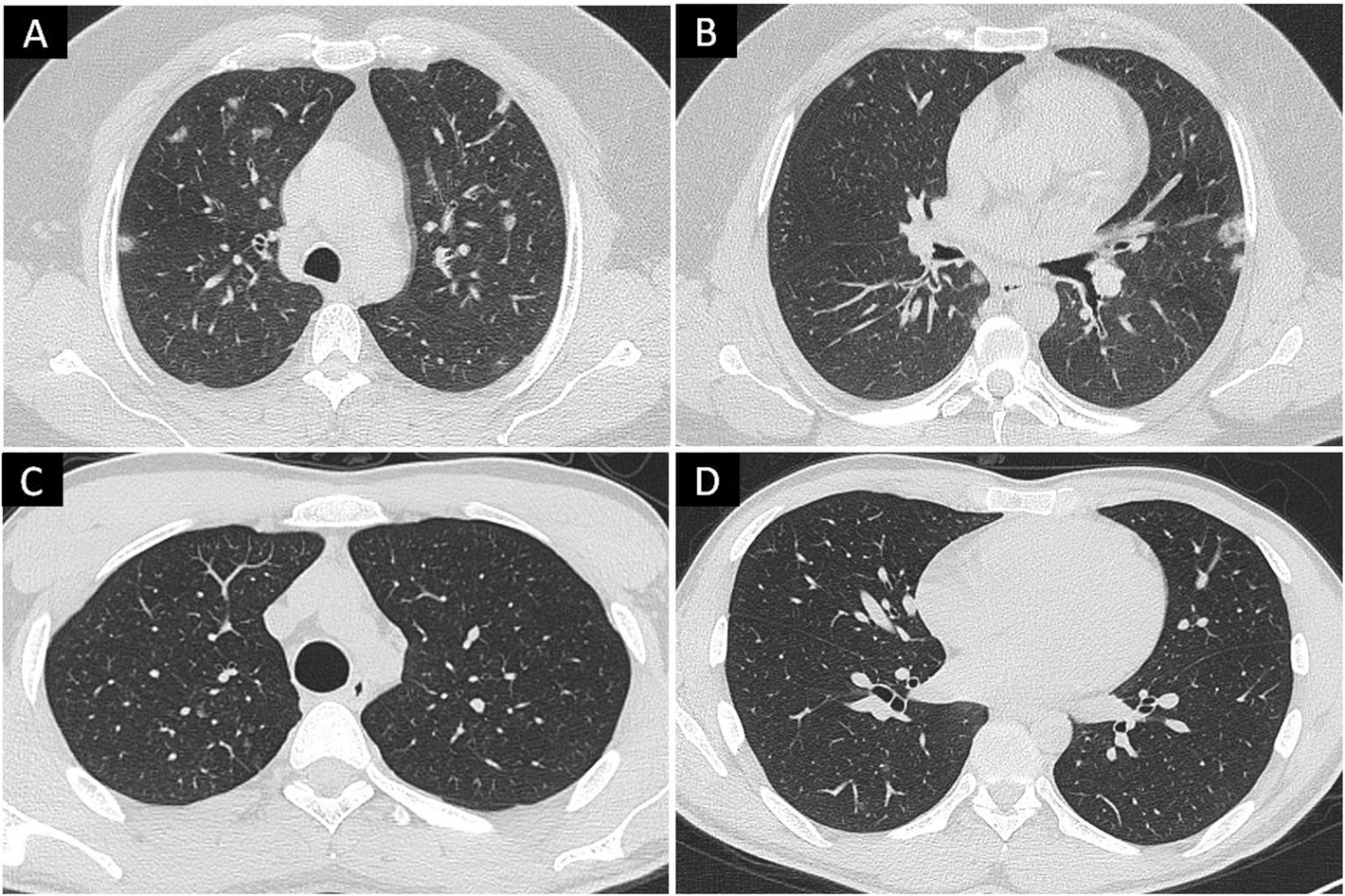
4**a**-4**b**: Moderate COVID-19, non-re-detectable positive patient. There are multiple pure and mixed ground-glass opacities under double lung pleural. The total scores of involved zones in the bilateral lung is 4. 4**c**-4**d**: Moderate COVID-19, re-detectable positive patient. There are multiple pure ground-glass opacities under upper and lower lobe pleural of the right lung. The total scores of involved zones in the bilateral lung is 2

The CT imaging features of the NRP and RP patients are shown in Table E5, and those imaging features with significant differences are presented in Table 5. Representative CT images from the NRP and RP patients are shown in Fig. 4. Among them, only the occurrence rates of mixed GGOs and mixed GGOs in the peripheral area were significantly different between the NRP and RP patients (all *P*< 0.05). Compared to NRP patients, negative rates of mixed GGOs (71.4%) and mixed GGOs in the peripheral area (71.4%) were more pronounced in the RP patients. Because most RP patients (85.7%) had moderate COVID-19 pneumonia, further analysisof the moderate COVID-19 patients revealed that RP patients with moderate COVID-19 had a smaller proportion of the left lung affected than the NRP patients (0.83±0.75 vs 1.53±0.69, *P*=0.024). In addition, total bilateral lung scores < 3 were more prevalent in RP patients with moderate COVID-19 than in NRP patients with moderate COVID-19 (66.7% vs.18.2%, *P*=0.030).

**Table 5:** CT imaging features of RP and NRP patients with COVID-19

Features	NRP patients(n=72)	RP patients(n=7)	*P*-value
Overall condition of lesions			0.682^c^
Normal^#^	10(13.9%)	1(14.3%)	
Single^#^	5(6.9%)	1(14.3%)	
Multiple^#^	57(79.2%)	5(71.4%)	
Total number of GGO inperipheral area			
Pure GGO	4.49±6.92	8.00±7.53	0.207^b^
Mixed GGO	4.46±6.86	2.43±5.59	0.451^b^
Total number of lesions			
Peripheral area	10.1±13.1	12.0±16.4	0.724^b^
Central area	1.44±2.90	4.14±11.0	0.103^b^
Total scores of involved lung zones			
Bilateral lung	4.93±4.04	3.00±3.65	0.227^b^
Right lung	2.81±2.45	2.00±2.31	0.407^b^
Left lung	2.12±1.86	1.00±1.41	0.125^b^
Number of lobes affected			
Right lung	1.82±1.24	1.43±0.98	0.353^b^
Left lung	1.33±0.82	0.71±0.76	0.059^b^
Total scores of bilateral lung category			0.064^a^
< 3 scores^#^	21(29.2%)	5(71.4%)	
≥3 scores^#^	51(70.8%)	2(28.6%)	
Pure GGO category			0.804^a^
Negative^#^	19(26.4%)	1(14.3%)	
Positive^#^	53(73.6%)	6(85.7%)	
Pure GGO in peripheral area category			0.746^a^
Negative^#^	20(27.8%)	1(14.3%)	
Positive^#^	52(72.2%)	6(85.7%)	
Mixed GGO category			0.018^a^*
Negative^#^	16(22.2%)	5(71.4%)	
Positive^#^	56(77. 8%)	2(28.6%)	
Mixed GGO in peripheral area category			0.032^a^*
Negative^#^	18(25.0%)	5(71.4%)	
Positive^#^	54(75.0%)	2(28.6%)	

Note: *Data with statistical significance. ^#^Results are measurements with corresponding ratio in parentheses, and the remainder results are mean value with standard deviation. *P*^a^: chi square test, *P*^b^: student’s *t* test, *P*^c^: Kruskal-Wallis *H* test, *GGO* Ground-glass opacity

### Baseline characteristics of RP patients at first hospitalization and readmission

All RP patients were readmitted to the hospital for further medical observation. The baseline characteristics of RP patients at first hospitalization and readmission are shown in Table 6. No patient had a fever or noticeable disease progression during readmission. The prevalence of fatigue and runny nose were decreased compared with the prior admission (14.3% vs 28.8 and 0.00% vs 14.3%, respectively). All RP patients had stable COVID-19 and their WBC count, lymphocyte count, lymphocyte rate, neutrophil count and neutrophil rate were wihtin the normal range. The total bilateral lung scores were reduced in RP patients at readmission compared with the scores at the first hospitalization (1.14±0.69 vs 3.00±3.65). Compare to the first hospitalization, RP patients had a shorter hospitalization period (*P*< 0.001) and fewer days between the onset of illness and last RNA negative conversion (*P*< 0.001) at readmission. In addition, because all discharged COVID-19 patients in our cohort required isolation at home, only 3 close contacts were followed up. As of April 30, 2020, all the close contacts were tested, and all were negative for SARS-CoV-2. No suspicious clinical symptoms were reported in those close contacts.

**Table 6:** Baseline characteristics of RP patients at first hospitalization and readmission

Features	First hospitalization (*n*=7)	Re-admission (*n*=7)	*P*-value
Signs			
Fever^#^	2(28.6%)	0(0.0%)	1.00^d^
Cough^#^	3(42.9%)	3(42.9%)	1.00^a^
Fatigue^#^	2(28.8%)	1(14.3%)	1.00^a^
Runny nose^#^	1(14.3%)	0(0.0%)	1.00^d^
Clinical types			1.00^a^
Mild^#^	1(14.3%)	1(14.3%)	
Moderate^#^	6(85.7%)	6(85.7%)	
Hospitalization days	18.3±5.35	6.57±1.62	< 0.001^b^*
Days of last RNA negative-conversion	15.3±5.38	3.57±1.90	< 0.001^b^*
WBC count (×10^9^/L)	7.07±3.29	6.42±1.51	0.642^b^
Lymphocyte count	1.22±0.38	1.56±0.39	0.124^b^
Lymphocyte rate (%)	21.0±10.4	25.3±6.78	0.378^b^
Neutrophil count(×10^9^/L)	5.14±3.23	3.97±1.20	0.385^b^
Neutrophil rate (%)	63.9±17.6	62.0±6.51	0.791^b^
Chest CT			1.00^a^
Normal^#^	1(14.3%)	1(14.3%)	
Abnormal^#^	6(85.7%)	6(85.7%)	
Total scores of bilateral lung	3.00±3.65	1.14±0.69	0.211^b^
Pure GGO category			1.00^a^
Negative^#^	1(14.3%)	1(14.3%)	
Positive^#^	6(85.7%)	6(85.7%)	
Pure GGO in peripheral area category			1.00^a^
Negative^#^	1(14.3%)	2(28.6%)	
Positive^#^	6(85.7%)	5(71.4%)	
Mixed GGO category			0.192^d^
Negative^#^	4(57.1%)	7(100%)	
Positive^#^	3(42.9%)	0(0.0%)	
Mixed GGO in peripheral area category			0.192^d^
Negative^#^	4(57.1%)	7(100%)	
Positive^#^	3(42.9%)	0(0.0%)	

Note: *Data with statistical significance. ^#^Results are measurements with the corresponding ratio in parentheses, and the remainingresults are mean value with standard deviation. *P*^a^: chi-square test, *P*^b^: student’s *t*-test, *P*^d^: Fisher’test. *WBC* White blood cell

## Discussion

In this study, we retrospectively analyzed data from onset to discharge for five independent COVID-19 groups from four different cities: re-detectable positivity and repeat hospitalization, and discharge were evaluated. We found that 26.6% of patients had atypical presentations of COVID-19, including asymptomatic, CT imaging-negative, and RP presentations. By comparing CT imaging and clinical characteristics between typical and atypical patients with COVID-19, 14 imaging features and 2 clinical characteristics were significantly different between symptomatic and asymptomatic COVID-19 patients. Five clinical characteristics were significantly different between CT-negative and CT-positive patients. Two imaging features were significantly different between RP and NRP patients with a moderate-severity COVID-19. Two clinical features were significantly different between the first hospitalization and readmission in RP patients.

Asymptomatic patients were first reported and shown to be contagious by Bai et al. [15]. In our study, half of the asymptomatic patients were mild cases, and the other half were moderate cases. In line with previous studies [8, 16], none of the asymptomatic patients developed severe COVID-19 pneumonia, and the hospitalization stays of asymptomatic patients were shorter than those of symptomatic patients. In a previous study, GGOs with a peripheral distribution were demonstrated to be the predominant feature of the CT findings in asymptomatic cases of COVID-19 [16]. Our findings also showed that asymptomatic patients had lower total bilateral lung scores and a smaller total number of GGOs in the peripheral area, indicating milder pneumonia in these patients. Since 80% of asymptomatic patients had a score of lower than 3 points and more than 73% of symptomatic patients had a score of higher than 3 points, we speculate that a total score of 3 can be used as a threshold.

The CT imaging-negative patients was significantly younger than the CT imaging-positive patients. Similarly, Ai et al. demonstrated that the positive predictive values and accuracy of chest CT in diagnosing COVID-19 were higher in patients ≥ 60 years than in patients < 60 years [17]. A recent study further revealed that most COVID-19-positive children had negative chest CT scans [18]. Due to the presence of CT imaging-negative patients, The use of CT as a diagnostic tool for COVID-19 is becoming controversial [19]. Multiple radiological organizations have suggested that CT should not be used to screen for or as a first-line test to diagnose COVID-19 [20–22]. Nevertheless, CT imaging plays an important role in the diagnosis and treatment of COVID-19 [3, 23]. In this study, we also found that half of the asymptomatic patients had negative CT imaging results. This evidence supports the statement that CT scanning is not a perfect tool for COVID-19 screening. However, the above evidence also indicates that the patient age can be one of the essential reference standards for chest CT usage: the use of CT may be more appropriate in elderly patients.

As an incerasing number of patients are cured and discharged from the hospital, RP patients with COVID-19 have become the focus of prevention. Several case reports noted that some COVID-19 patients test positive days after recovery [7, 24]; most cases were of mild and moderate severity [25]. Consistent with previous research, our findings demonstrated that no severe patient with COVID-19 developed RP. Moreover, most RP patients had a total lung involvement score of less than 3, which is similar in asymptomatic patients, confirming that the total lung involvement score is an effective indicator for classifying typical and atypical COVID-19 patients. The contagiousness of RP patients is still unclear, and some believe that the positive PCR results are from specific gene fragments that have no contagiousness [26, 27]. In our study, we found that all close contacts with RP patients were negative for SARS-CoV-2. However, it is worth noting that some RP patients previously had a high viral load of SARS-CoV-2 nucleic acid [28]. Therefore, COVID-19 prevention should not end at discharge, and a proper extension of the quarantine time after discharge may be required. Fortunately, our study suggests that the number of days sincer last RNA negative conversion are much fewer in RP patients when they are readmitted to the hospital than at their fist admission.

There are several limitations to this study. First, the asymptomatic population may be underestimated since it is difficult to estimate how many people become infected without showing symptoms. In this study, only close contacts of the COVID-19 patients were tracked. Population-wide testing is needed in further studies. Second, antibody tests such as IgG and IgM tests were missing in our study since this technology had not been utilized in the partcipant hospitals at that time. Further research that incorporates blood antibody tests may better detect false-negative PCR cases.

## Conclusions

In conclusion, we analyzed the atypical presentations of COVID-19 from onset to readmission, including patients with asymptomatic, CT imaging negative, and RP presentations. Significant CT imaging and clinical feature differences were found between atypical and typical COVID-19 patients for all three presentation, which helps provide complementary information for the effective management of COVID-19.

## Supplementary Information

**Additional file 1.**

## Data Availability

The data cohorts used and/or analyzed during the present study are available from the corresponding author on reasonable request.

## Abbreviations

COVID-19: Coronavirus disease 2019
GGO: Ground-glass opacity
RT-PCR: Reverse transcription polymerase chain reaction
WBC: White blood cell
AUC: Area under the curve
SARS: Severe acute respiratory syndrome
MERS: Middle east respiratory syndrome
