# Supplementary material for: Atypical presentations of coronavirus disease 2019 (COVID-19) from onset to readmission
Source: BMC Infect Dis. 2021 Jan 29;21:127. doi: 10.1186/s12879-020-05751-8 (PMC7844102; doi:10.1186/s12879-020-05751-8)
Supplement: Supplementary file 1 — Additional file 1. [file 12879_2020_5751_MOESM1_ESM.docx]

**Supplementary Materials**

**Table of Contents：**

[**1.**](#_Toc35425435) [**CT imaging protocols and acquisition parameters (Table E1)..........................2**](#_Toc35425436)

[**2. Definition of radiological semantic features (Table E2 and E3)........................4**](#_Toc35425437)

[**3.**](#_Toc35425438) [**CT imaging features of symptomatic and asymptomatic patients with COVID-19 (TableE4)....................................................................................................8**](#_CT_imaging_features)

[**4. CT imaging features of RP and NRP patients with COVID-19 (TableE5).....13**](#_CT_imaging_features_1)

# CT imaging protocols and acquisition parameters (Table E1)

We selected patient images that were acquired on various models of multi-row spiral CT scans from GE, Scenaria and Philips scanners. Table E1 shows the CT imaging protocols and acquisition parameters of each medical institutions.

**Table E1: CT imaging protocols and acquisition parameters of each medical institutions**

| CT imaging protocols and acquisition parameters | Yongzhou people's hospital, Hunan province | The first affiliated hospital of Shantou university medical college | Shantou central hospital, Guangdong province | Huizhou central hospital, Guangdong province | Meizhou people’s hospital, Guangdong province |
| --- | --- | --- | --- | --- | --- |
| CT scanner | 64-section scanner (SCENARIA 64 CT, Hitachi Medical, Japan). | 64-section scanner (GE CT Discovery 750 HD) | 64-section scanner (Ingenuity CT, PHILIPS) | 64-section scanner (Ingenuity CT, PHILIPS) | 64-slice multidetector spiral CT (Somatom Definition AS, Siemens, Guangdong, China) |
| Tube voltage (kV) | 130 | 120 | 120 | 120 | 120 |
| Automatic tube current (mA) | 180-350 | 320 | 132-459 | 400 -500 | 150 |
| Iterative reconstruction technique | + | + | + | + | + |
| Detector (mm) | 64 | 40 | 64 | 64 | 128 |
| Rotation time (s) | 0.5 | 0.5 | 0.75 | 0.5 | 0.5 |
| Section thickness (mm) | 5 | 5 | 5 | 5 | 1.5 |
| Collimation | 0.6 | 0.6 | 0.625 | 0.625 | 0.6 |
| Pitch | 0.99 | 1.5 | 0.98 | 0.9 | 1.2 |
| Matrix | 512 × 512 | 512 × 512 | 512 × 512 | 512 × 512 | 512 × 512 |

# Definition of radiological semantic features (Table E2 and E3)

Table E2 presents definitions of radiological feature, which could benefit to understand each feature in CT images and read smoothly. These definitions are cited from study of Hansell DM, et al in 2008 (1).

**Table E2: Definition of CT qualitative imaging features**

| CT qualitative imaging features | Definition |
| --- | --- |
| Ground glass opacities(GGO) | It appears as hazy increased opacity of lung, with preservation of bronchial and vascular margins. |
| Pure GGO | Equal to GGO |
| Pure GGO in peripheral area | The pure GGO in the outer third of the lung |
| Pure GGO in central area | The pure GGO in the inner two thirds of the lung |
| Consolidation | Consolidation appears as a homogeneous increase in pulmonary parenchymal attenuation that obscures the margins of vessels and airway walls. |
| Mixed GGO | The combination of GGO and consolidation |
| Mixed GGO in peripheral area | The mixed GGO in the outer third of the lung |
| Mixed GGO in central area | The mixed GGO in the inner two thirds of the lung |
| Emphysema | The CT appearance of emphysema consists of focal areas or regions of low attenuation, usually without visible walls. |
| Air bronchogram | An air bronchogram is a pattern of air-filled (low-attenuation) bronchi on a back-ground of opaque (high-attenuation) air-less lung. |
| Interlobular septal thickening | On CT scans, disease affecting one of the components of the septa may be responsible for thickening and so render septa visible. |
| Crazy-paving pattern | This pattern appears as thickened interlobular septa and intralobular lines superimposed on a background of ground-glass opacity, resembling irregularly shaped paving stones. |
| Tree-in-bud sign | The tree-in-bud pattern represents centrilobular branching structures that resemble a budding tree. |
| Cavity | A cavity is a gas-filled space, seen as a lucency or low-attenuation area, within pulmonary consolidation, a mass, or a nodule. |
| Lymphadenopathy | By common usage, the term lymphadenopathy is usually restricted to enlargement, due to any cause, of the lymph nodes. Somewhat arbitrary thresholds for the upper limit of normal of 1 cm in short-axis diameter for mediastinal nodes and 3 mm for most hilar nodes have been reported. |
| Offending vessel augmentation in lesions | The vessel associated with lesions is enlarged in CT image. |
| Overall condition of lesions | The total number of lesion in the lung, including 0,1 and ≥ 2, corresponding to normal, single and multiple. |
| Pleural thickening | The involved pleura is thickening as elevated flat or nodular lesions. |
| Pleural traction | Pleural traction was defined as depression areas of pleural thickening |
| Pleural effusions | The CT appearance of pleural effusions consists of fluid in the pleural space. |
| Bronchial wall thickening | Bronchial wall thickness was defined as bronchovascular interstitial thickening |
| Linear opacities | Linear opacities was defined as continuous thickening of peribronchial area |

**Table E3: Definition of CT quantitative imaging features**

| CT quantitative imaging features | Definition |
| --- | --- |
| Total number of pure GGO | The total number of pure GGO in the bilateral lung |
| Number of pure GGO in peripheral area | The total number of pure GGO in the outer third of the lung |
| Number of pure GGO in central area | The total number of pure GGO in the inner two thirds of the lung |
| Total number of mixed GGO | The total number of mixed GGO in the bilateral lung |
| Number of mixed GGO in peripheral area | The total number of mixed GGO in the outer third of the lung |
| Number of mixed GGO in central area | The total number of mixed GGO in the inner two thirds of the lung |
| Total number of consolidation | The total number of consolidation in the bilateral lung |
| Total number of solid nodules | The total number of solid nodules in the bilateral lung |
| Total number of lesions in peripheral area | The total number of pure GGO and mixed GGO in the outer third of the lung |
| Total number of lesions in central area | The total number of pure GGO and mixed GGO in the inner two thirds of the lung |
| Total number of lesions in both peripheral and central area | The total number of pure GGO and mixed GGO in both inner two thirds and outer third of the lung |
| Lesion sizes < 1cm | The total number of pure GGO and mixed GGO with diameter < 1cm in the lung |
| Lesion sizes: 1cm to 3cm | The total number of pure GGO and mixed GGO with 1cm ≤ diameter ≤3 cm in the lung |
| Lesion sizes : 3cm to half of segments | The total number of pure GGO and mixed GGO with 3< diameter≤ half of segments in the lung |
| Lesion sizes:＞half of segments | The total number of pure GGO and mixed GGO with diameter > half of segments in the lung |
| Total scores of bilateral lung | The scores was combined for all involved lobes in the bilateral lung |
| Total scores of right lung | The scores was combined for all involved lobes in the right lung |
| Total scores of left lung | The scores was combined for all involved lobes in the left lung |
| Total scores of bilateral upper lobes lung | The scores was combined for all involved lobes in the bilateral upper lobes lung |
| Total scores of bilateral lower lobes lung | The scores was combined for all involved lobes in the bilateral lower lobes lung |
| Number of right lung lobes affected | The total number of involved lobes in the right lung |
| Number of left lung lobes affected | The total number of involved lobes in the left lung |
| Total number of bilateral lung involved segments | The total number of involved lobes in the bilateral lung |
| Total number of bilateral upper lobes involved segments | The total number of involved lobes in the bilateral upper lobes |
| Total number of bilateral lower lobes involved segments | The total number of involved lobes in the bilateral lower lobes |

# CT imaging features of symptomatic and asymptomatic COVID-19 patients

| **Table E4 CT imaging features of symptomatic and asymptomatic COVID-19 patients** | | | |
| --- | --- | --- | --- |
| Features | Symptomatic (n=69) | Asymptomatic (n=10) | *P*-value |
| Number of pure GGO |  |  |  |
| Total | 7.39±11.5 | 1.30±2.36 | 0.100^b^ |
| Peripheral area | 5.36±7.28 | 0.90±2.18 | <0.001^b^* |
| Central area | 1.96±4.22 | 0.40±0.97 | 0.252^b^ |
| Number of mixed GGO |  |  |  |
| Total | 6.49±9.71 | 1.20±2.82 | 0.092^b^ |
| Peripheral area | 4.83±7.05 | 0.50±0.85 | <0.001^b^* |
| Central area | 1.77±3.30 | 0.70±2.21 | 0.326^b^ |
| Total number of consolidation and solid nodules | |  |  |
| Consolidation | 0.64±1.69 | 0.00±0.00 | 0.239^b^ |
| Pure solid nodules | 0.13±0.38 | 0.00±0.00 | 0.284^b^ |
| Total number of lesions |  |  |  |
| Peripheral area | 11.6±13.8 | 1.40±2.22 | <0.001^b^* |
| Central area | 1.81±4.41 | 0.80±1.93 | 0.478^b^ |
| Both peripheral and central area | 2.28±5.54 | 0.30±0.67 | 0.266^b^ |
| Lesion sizes |  |  |  |
| < 1cm | 3.42±5.16 | 0.40±0.52 | <0.001^b^* |
| 1cm to 3cm | 8.72±14.5 | 1.80±2.78 | 0.139^b^ |
| 3cm to half of segments | 2.55±5.07 | 0.00±0.00 | 0.118^b^ |
| ＞half of segments | 0.91±2.15 | 0.30±0.67 | 0.375^b^ |
| Total scores of involved lung zones | |  |  |
| Bilateral lung | 5.26±4.01 | 1.30±1.83 | <0.001^b^* |
| Right lung | 3.01±2.45 | 0.80±1.23 | <0.001^b^* |
| Left lung | 2.25±1.86 | 0.50±0.71 | <0.001^b^* |
| Bilateral upper lobes | 1.74±1.47 | 0.60±0.70 | 0.019^b^* |
| Bilateral lower lobes | 2.71±2.17 | 0.50±0.85 | <0.001^b^* |
| Number of lobes affected |  |  |  |
| Right lung | 1.93±1.15 | 0.80±1.23 | 0.005^b^* |
| Left lung | 1.39±0.79 | 0.50±0.71 | 0.001^b^* |
| Total number of involved lung segments | |  |  |
| Bilateral lung | 3.32±1.78 | 1.30±1.83 | 0.001^b^* |
| Bilateral upper lobes | 1.32±0.88 | 0.60±0.70 | 0.016^b^* |
| Bilateral lower lobes | 1.48±0.78 | 0.50±0.85 | <0.001^b^* |
| Overall condition of lesions | |  | <0.001^c^* |
| Normal^#^ | 6(8.7%) | 5(50.0%) |  |
| Single^#^ | 4(5.8%) | 2(20.0%) |  |
| Multiple^#^ | 59(85.5%) | 3(30.0%) |  |
| Total scores of bilateral lung category | |  | 0.002^a^* |
| <3 scores^#^ | 18(26.1%) | 8(80.0%) |  |
| ≥3 scores^#^ | 51(73.9%) | 2(20.0%) |  |
| Pure GGO category |  |  | 0.021^a^* |
| Negative^#^ | 14(20.3%) | 6(60.0%) |  |
| Positive^#^ | 55(79.7%) | 4(40.0%) |  |
| Pure GGO in peripheral area category | |  | 0.003^a*^ |
| Negative^#^ | 14(20.3%) | 7(70.0%) |  |
| Positive^#^ | 55(79.7%) | 3(30.0%) |  |
| Pure GGO in central area category | |  | 0.285^a^ |
| Negative^#^ | 39(56.5%) | 8(80.0%) |  |
| Positive^#^ | 30(43.5%) | 2(20.0%) |  |
| Mixed GGO category |  |  | 0.003^a^* |
| Negative^#^ | 14(20.3%) | 7(70.0%) |  |
| Positive^#^ | 55(79.7%) | 3(30.0%) |  |
| Mixed GGO in peripheral area category | |  | 0.008^a^* |
| Negative^#^ | 16(23.2%) | 7(70.0%) |  |
| Positive^#^ | 53(76.8%) | 3(30.0%) |  |
| Mixed GGO in central area category | |  | 0.055^a^ |
| Negative^#^ | 36(52.2%) | 9(90.0%) |  |
| Positive^#^ | 33(47.8%) | 1(10.0%) |  |
| Pleural thickening |  |  | 0.526^a^ |
| Negative^#^ | 37(53.6%) | 7(70.0%) |  |
| Positive^#^ | 32(46.4%) | 3(30.0%) |  |
| Linear opacities |  |  | 0.056^a^ |
| Negative^#^ | 29(42.0%) | 8(80.0%) |  |
| Positive^#^ | 40(58.0%) | 2(20.0%) |  |
| Emphysema |  |  | 0.569^a^ |
| Negative^#^ | 64(92.8%) | 9(90.0%) |  |
| Positive^#^ | 5(7.20%) | 1(10.0%) |  |
| Air bronchogram |  |  | 0.069^a^ |
| Negative^#^ | 30(43.5%) | 8(80.0%) |  |
| Positive^#^ | 39(56.5%) | 2(20.0%) |  |
| Bronchial wall thickening |  |  | 0.010^a^* |
| Negative^#^ | 28(40.6%) | 9(90.0%) |  |
| Positive^#^ | 41(59.4%) | 1(10.0%) |  |
| Interlobular septal thickening | |  | 0.016^a^* |
| Negative^#^ | 30(43.5%) | 9(90.0%) |  |
| Positive^#^ | 39(56.5%) | 1(10.0%) |  |
| Crazy paving pattern |  |  | 0.118^a^ |
| Negative^#^ | 33(47.8%) | 8(80.0%) |  |
| Positive^#^ | 36(52.2%) | 2(20.0%) |  |
| Tree in bud |  |  | 0.496^d^ |
| Negative^#^ | 60(87.0%) | 10(100%) |  |
| Positive^#^ | 9(13.0%) | 0(0.0%) |  |
| Cavity |  |  | 0.239^a^ |
| Negative^#^ | 68(98.6%) | 9(90.0%) |  |
| Positive^#^ | 1(1.40%) | 1(10.0%) |  |
| Offending vessel augmentation in lesions | |  | 0.002^a^* |
| Negative^#^ | 18(26.1%) | 8(80.0%) |  |
| Positive^#^ | 51(73.9%) | 2(20.0%) |  |
| Note: *Data with statistical significance. ^#^Results are measurements with corresponding ratio in parentheses, and the remainder results are mean value with standard deviation. *P*^a^: chi square test, *P*^b^: student’s *t* test, *P*^c^: Kruskal-Wallis *H* test, *P*^d^: Fisher' test, GGO= Ground-glass opacity. | | | |

# CT imaging features of RP and NRP patients with COVID-19

| **Table E5 CT imaging features of RP and NRP patients with COVID-19** | | | |
| --- | --- | --- | --- |
| Features | NRP patients (n=72) | RP patients(n=7) | *P*-value |
| Number of pure GGO |  |  |  |
| Total | 6.25±10.7 | 10.4±13.3 | 0.338^b^ |
| Peripheral area | 4.49±6.92 | 8.00±7.53 | 0.207^b^ |
| Central area | 1.69±3.74 | 2.43±6.43 | 0.645^b^ |
| Number of mixed GGO |  |  |  |
| Total | 5.96±9.19 | 4.43±10.9 | 0.680^b^ |
| Peripheral area | 4.46±6.86 | 2.43±5.59 | 0.451^b^ |
| Central area | 1.60±2.97 | 2.00±5.29 | 0.752^b^ |
| Total number of consolidation and solid nodules | |  |  |
| Consolidation | 0.46±1.10 | 1.57±4.16 | 0.506^b^ |
| Pure solid nodules | 0.12±0.37 | 0.00±0.00 | 0.381^b^ |
| Total number of lesions |  |  |  |
| Peripheral area | 10.1±13.1 | 12.0±16.4 | 0.724^b^ |
| Central area | 1.44±2.90 | 4.14±11.0 | 0.103^b^ |
| Both peripheral and central area | 2.03±5.28 | 2.00±4.86 | 0.989^b^ |
| Lesion sizes |  |  |  |
| < 1cm | 2.86±4.82 | 4.86±5.98 | 0.309^b^ |
| 1cm to 3cm | 7.47±12.72 | 11.7±23.3 | 0.441^b^ |
| 3cm to half of segments | 2.31±4.94 | 1.43±3.36 | 0.648^b^ |
| ＞half of segments | 0.92±2.11 | 0.00±0.00 | 0.256^b^ |
| Total scores of involved lung zones | |  |  |
| Bilateral lung | 4.93±4.04 | 3.00±3.65 | 0.227^b^ |
| Right lung | 2.81±2.45 | 2.00±2.31 | 0.407^b^ |
| Left lung | 2.12±1.86 | 1.00±1.41 | 0.125^b^ |
| Bilateral upper lobes | 1.67±1.46 | 0.86±1.07 | 0.159^b^ |
| Bilateral lower lobes | 2.51±2.14 | 1.57±2.51 | 0.277^b^ |
| Number of lobes affected |  |  |  |
| Right lung | 1.82±1.24 | 1.43±0.98 | 0.353^b^ |
| Left lung | 1.33±0.82 | 0.71±0.76 | 0.059^b^ |
| Total number of involved lung segments | |  |  |
| Bilateral lung | 3.15±1.91 | 2.14±1.57 | 0.180^b^ |
| Bilateral upper lobes | 1.28±0.89 | 0.71±0.76 | 0.111^b^ |
| Bilateral lower lobes | 1.38±0.85 | 1.14±0.90 | 0.493^b^ |
| Overall condition of lesions | |  | 0.682^c^ |
| Normal^#^ | 10(13.9 %) | 1(14.3 %) |  |
| Single^#^ | 5(6.9 %) | 1(14.3 %) |  |
| Multiple^#^ | 57(79.2 %) | 5(71.4 %) |  |
| Total scores of bilateral lung category | |  | 0.064^a^ |
| <3 scores^#^ | 21(29.2%) | 5(71.4%) |  |
| ≥3 scores^#^ | 51(70.8%) | 2(28.6%) |  |
| Pure GGO category |  |  | 0.804^a^ |
| Negative^#^ | 19(26.4%) | 1(14.3%) |  |
| Positive^#^ | 53(73.6%) | 6(85.7%) |  |
| Pure GGO in peripheral area category | |  | 0.746^a^ |
| Negative^#^ | 20(27.8%) | 1(14.3%) |  |
| Positive^#^ | 52(72.2%) | 6(85.7%) |  |
| Pure GGO in central area category | |  | 0.281^a^ |
| Negative^#^ | 41(56.9%) | 6(85.7%) |  |
| Positive^#^ | 31(43.1%) | 1(14.3%) |  |
| Mixed GGO category |  |  | 0.018^a^* |
| Negative^#^ | 16(22.2%) | 5(71.4%) |  |
| Positive^#^ | 56(77. 8%) | 2(28.6%) |  |
| Mixed GGO in peripheral area category | |  | 0.032^a^* |
| Negative^#^ | 18(25.0%) | 5(71.4%) |  |
| Positive^#^ | 54(75.0%) | 2(28.6%) |  |
| Mixed GGO in central area category | |  | 0.226^a^ |
| Negative^#^ | 39(54.2%) | 6(85.7%) |  |
| Positive^#^ | 33(45.8%) | 1(14.3%) |  |
| Pleural thickening |  |  | 0.632^a^ |
| Negative^#^ | 39(54.2%) | 5(71.4%) |  |
| Positive^#^ | 33(45.8%) | 2(28.6%) |  |
| Linear opacities |  |  | 0.332^a^ |
| Negative^#^ | 32(44.4%) | 5(71.4%) |  |
| Positive^#^ | 40(55.6%) | 2(28.6%) |  |
| Emphysema |  |  |  |
| Negative^#^ | 66(91.7%) | 7(100%) | 1.00^d^ |
| Positive^#^ | 6(8.30%) | 0(0.0%) |  |
| Air bronchogram |  |  | 0.091^a^ |
| Negative^#^ | 32(44.4%) | 6(85.7%) |  |
| Positive^#^ | 40(55.6%) | 1(14.3%) |  |
| Bronchial wall thickening |  |  | 0.078^a^ |
| Negative^#^ | 31(43.1%) | 6(85.7%) |  |
| Positive^#^ | 41(56.9%) | 1(14.3%) |  |
| Interlobular septal thickening | |  | 0.105^a^ |
| Negative^#^ | 33(45.8%) | 6(85.7%) |  |
| Positive^#^ | 39(54.2%) | 1(14.3%) |  |
| Crazy paving pattern |  |  | 0.139^a^ |
| Negative^#^ | 35(48.6%) | 6(85.7%) |  |
| Positive^#^ | 37(51.4%) | 1(14.3%) |  |
| Tree in bud |  |  | 0.586^a^ |
| Negative^#^ | 64(88.9%) | 6(85.7%) |  |
| Positive^#^ | 8(11.1%) | 1(14.3%) |  |
| Cavity |  |  | 1.00^d^ |
| Negative^#^ | 70(97.2%) | 7(100%) |  |
| Positive^#^ | 2(2.78%) | 0(0.0%) |  |
| Offending vessel augmentation in lesions | |  | 0.064^a^ |
| Negative^#^ | 21(29.2%) | 5(71.4%) |  |
| Positive^#^ | 51(70.8%) | 2(28.6%) |  |
| Note: *Data with statistical significance. ^#^Results are measurements with corresponding ratio in parentheses, and the remainder results are mean value with standard deviation. *P*^a^: chi square test, *P*^b^: student’s *t* test, *P*^c^: Kruskal-Wallis *H* test, *P^d^*: Fisher' test, GGO= Ground-glass opacity. | | | |

Reference

1. Hansell DM, Bankier AA, MacMahon H, McLoud TC, Müller NL, Remy J. Fleischner Society: glossary of terms for thoracic imaging. Radiology. 2008; 246(3):697-722.
